# Comparison of the Effectiveness Differences between Western and Chinese Medicinal Ointments against Eczema

**DOI:** 10.3390/ph18091248

**Published:** 2025-08-22

**Authors:** Siu Kan Law, Yanping Wang, Xiao Xiao Wu

**Affiliations:** 1 Medical Laboratory of Shenzhen Luohu Hospital Group, Shenzhen 518000, China; 2 School of Nursing and Health Sciences, Hong Kong Metropolitan University, Hong Kong SAR, China; ypwang@hkmu.edu.hk

**Keywords:** Western medicine, Chinese medicine, ointment, eczema

## Abstract

Eczema is the most common skin disease among Hong Kong’s adults and children, affecting an estimated 30% of the total population. Western and Chinese medicinal ointments are the usual treatment for eczema. Conventional Western medicinal ointments are topical corticosteroids and non-steroidal agents. Eczema skin products include “Aveeno Parabens Lotion”, “Cerave Moisturizing Cream”, and “Cetaphil Lotion”. However, these are not a long-term solution for managing significant erythema. Chinese medicinal ointments are based on adjusting the formula, including the ingredients and amount, to address an individual’s skin condition and other factors that may be worsening symptoms. This approach aims to regulate the immune system and make it less reactive to environmental and food allergies. This approach is mainly for local topical use. The ingredients of eczema skin products should include *Coptis chinensis* Franch, *Phellodendron chinense* Schneid, *Angelica sinensis* (Oliv.) Diels, *Rehmannia glutinosa* Libosch, *Curcuma longa* L., and sesame oil. Chinese medicinal ointments are natural ingredients, personalized formulas, and concerned with holistic healing, while Western medicinal ointments provide fast-acting relief, targeted action, and a standardized dosage. Methods: Nine electronic databases, such as WanFang Data, PubMed, Science Direct, Scopus, Web of Science, Springer Link, SciFinder, and the China National Knowledge Infrastructure (CNKI), were searched mainly within the past twenty years and without any language restrictions. The inclusion criteria were the keywords “Western medicine and ointment”, “Chinese medicine and ointment”, and “Western and Chinese medicines and ointment”. Differences in effectiveness between Western and Chinese ointments were evaluated to determine if they had functions against eczema. This review included an analysis and summary of all relevant papers. Results: Western medicinal ointments are topical corticosteroids, and they exert their pharmacological activities via many mechanisms, including anti-inflammatory, immunosuppressive, antiproliferative, and vasoconstrictive effects on eczema. Similarly, Chinese medicinal ointments have the same pharmacological functions, but they may focus on the immune system for the treatment of inflammatory and skin conditions, including erythema, edema, dryness, desquamation, and callus exfoliation. Conclusion: Based on the clinical research, the effectiveness rate of integrated Chinese and Western medicines was 88%, which was greater than the 70% rate for using Western medicine alone to treat eczema. Western and Chinese medicinal ointments have different active ingredients with advantages and disadvantages for eczema or when acting as skin care products. The most important thing is knowing “How” to use Western and Chinese medicinal ointments properly, especially for some formulations of Chinese ointments. It may be beneficial to consider the pharmacokinetic studies of herbal ingredients, which offer personalized formulas tailored to individual body constitutions and conditions, as well as to emphasize holistic healing, addressing both symptoms and underlying imbalances in the body. Much more work needs to be carried out, such as safety assessments of these ointments for use as skin care products for eczema.

## 1. Introduction

Eczema is a chronic skin disease that has risen proportionally among Hong Kong’s adults and children, affecting nearly 30% of the total population [1]. It is an itchy rash beginning with the conditions of dry, itchy skin, which leads to a rash because of patients scratching or rubbing the skin [2]. The pathogenesis of eczema includes social, biogenic, and anthropogenic environmental causes, as well as nutritional contributions [3]. Social activities are associated with psychosocial stress, such as personal depression, illness identity, anger, frustration, and psychosomatic states, and increased eczema symptom severity [4]. Eczema may be related to genetic problems with skin barrier function, but the effective targeting of the pathomechanism involved remains to be investigated [5]. An anthropogenic environment is associated with outdoor air pollutants, such as particulate matter (PM), volatile organic compounds (VOCs), gaseous compounds, and heavy metals. Exposure to indoor pollutants, including tobacco smoke and fungal molds, may also increase the risk of eczema [6]. Food nutrition can trigger rapid, immunoglobulin E-mediated hypersensitivity reactions or cause late eczematous reactions [7].

Generally, patient history and clinical or physical examinations are used in the diagnosis of eczema. It is quite common during infancy, and babies and children who are prone to it usually outgrow it as they age, but it is more likely to persist during the teenage years. Adult eczema is associated with allergies starting at 3 months of age. It may be further treated with the help of medicines [8]. There is a wide range of clinical presentations of eczema that vary according to patient factors, including age, skin type, ethnicity, and other comorbid conditions [9]. Physical examinations include serum IgE, potassium hydroxide (KOH) preparation, patch testing, and/or genetic testing. These may be helpful guidelines for ruling out other conditions of the skin [10], and they involve the assessment of the diagnostic features, extent, and severity of eczema, as well as the clinical evidence of secondary infection.

Based on the American Academy of Dermatology guidelines, the diagnostic features of eczema have four stages, which are summarized below (Table 1).

The possible treatment of eczema includes moisturizing the skin to repair and maintain the skin barrier, as well as to prevent the dehydration of the skin layer. Sometimes, it is also necessary to reduce itching and inflammation by managing the skin and decreasing. Western and Chinese ointments are the external treatments used for eczema. Conventional Western medicinal ointments are topical corticosteroids, including hydrocortisone, desonide, clobetasol propionate, fluocinolone, mometasone, fluticasone, aclometasone, prednicarbate, and non-steroidal agents such as prednisone, prednisolone, triamcinolone acetonide, and crisaborole. Chinese medicinal ointments are usually organic and natural, originating from herbal plants, for example, *Coptis chinensis* Franch, *Phellodendron chinense* Schneid., *Angelica sinensis* (Oliv.) Diels, *Rehmannia glutinosa* Libosch, *Curcuma longa* L., and sesame oil. These are novel medications used to treat eczema because of their anti-inflammatory and anti-allergic activities [12]. However, the best form of treatment is focusing on the body’s immune system to prevent inflammatory changes.

This review article aims to explore the active ingredients, mechanisms, advantages, and disadvantages of commonly used Western or Chinese medicinal ointments, as well as discuss some clinical examples of Western and Chinese medicinal ointments used in the treatment of eczema. The most important thing is knowing “how” to apply Western and Chinese ointments correctly to fight against eczema.

## 2. Western Medicines and Chinese Medicinal Ointments

### 2.1. Western Medicinal Ointments

Western medicinal ointments usually consist of topical corticosteroids or non-steroidal components, which have different chemical structures with specific pharmacological functions and mechanisms of action, as well as specific clinical outcomes (Table 2 and Table 3).

### 2.2. Chinese Medicinal Ointments

Chinese ointments are usually organic and natural, originating from herbal plants, for example, *Coptis chinensis* Franch., *Phellodendron chinense* Schneid., *Angelica sinensis* (Oliv.) Diels, *Rehmannia glutinosa* Libosch, *Curcuma longa* L., and sesame oil. Similarly, Chinese medicinal plants have different active ingredients and chemical structures with pharmacological functions and mechanisms, and related clinical studies are summarized in Table 4 and Table 5.

## 3. Limitations

### 3.1. Western Medicinal Ointments

There are some drawbacks to using topical corticosteroids or non-steroidal components in Western medicinal ointments for treating eczema.

Typically, less than 2% of topical corticosteroids (e.g., hydrocortisone) are absorbed into systemic circulation after a single application for more than 1 day [77]. There have been no immediate severe reactions to desonide, and most reported events were classified as mild and local reactions [78]. Long-term use of clobetasol propionate may cause photosensitivity, Cushing’s syndrome, allergic contact dermatitis, osteonecrosis, hypopigmentation, steroid acne, and skin atrophy [79]. Fluocinolone cream can aggravate and prolong telangiectasia, and most patients can experience severe rebound inflammation, edema, and acute pustular rash after stopping treatment [80]. Skin burning, stinging, folliculitis, dryness, an acne-like rash, and signs of skin atrophy are recorded as side effects of using Mometasone cream [25]. Fluticasone cream may cause adrenal gland problems, darkening of the skin, diarrhea, dizziness, fainting, loss of appetite, depression, nausea, rash, unusual tiredness or weakness, or vomiting [81]. Alclometasone is slightly stronger than hydrocortisone, but it may lead to breathing problems, wheezing, a fast heartbeat, fever, or general sickness [82]. Prednicarbate leads to the development of allergic contact dermatitis after using it for several weeks [83]. Indeed, Harvey et al. reported on the long-term safety of topical corticosteroids in atopic dermatitis. Skin became thin when these treatments were used intermittently for up to five years [84]. These topical corticosteroids may cause a range of systemic side effects, especially in infants and elderly patients, such as suppression of the hypothalamic–pituitary–adrenal axis, iatrogenic Cushing syndrome, slowed growth in infants and children, glaucoma and visual loss, avascular necrosis of the femoral head, and severe disseminated cytomegalovirus infection leading to death in infants [85].

Some common side effects of non-steroidals include pruritus, morbilliform rashes, urticaria, and photosensitivity [86]. Side effects of prednisone include hyperglycemia, insomnia, increased appetite, hypertension, osteoporosis, edema, adrenal suppression, cataracts, and delayed wound healing [87]. Prednisolone might significantly reduce leucocyte infiltration and activation, as well as modify the magnitude of the cutaneous latency [88]. Triamcinolone acetonide is associated with adverse effects, including itchiness, burning, irritation, or skin drying [89]. Crisaborole might cause pain, particularly stinging and skin burning [90]. Despite these risks, corticosteroids remain first-line treatments due to their rapid anti-inflammatory effects.

### 3.2. Chinese Medicinal Ointments

Several active ingredients for Chinese medicinal ointments, for example, “Berberine”, increase the number of melanocytes in the subepidermal layer and enhance the number of melanin-containing dendrites in melanocytes, causing the skin to darken [91]. Phellodendrine might decrease the body’s ability to break down cyclosporine and increase the chances of side effects, such as “Herbal-Drug interaction”; these components cannot be used concurrently and may cause skin allergies [92]. Ligustilide is an unstable, promising natural senescent cytotoxic compound, though it may produce a toxic effect on the skin [93]. Since catalpol may have a short half-life in the body and high water solubility, it struggles to pass through the blood–brain barrier, and its clinical application is severely limited [94]. Aucuboside may have toxic side effects and limited efficacy [65]. Acteoside can increase blood pressure and blood lipids in clinical studies (Table 6) [95].

## 4. Discussion

### 4.1. Basic Pathogenesis of Eczema

Atopic dermatitis is a chronic, relapsing inflammatory skin disease with a complex pathogenesis that includes (a) genetic factors, (b) epidermal barrier, (c) immune mechanism dysfunction, and (d) environmental triggers [96]. The following findings were made in this study:

(a) Eczema has been characterized by reference to loci on chromosomes 1q21, 3q21, 16q, 17q25, and 3p26, most notably 1q21, which contains a family of epithelial-related genes called the epidermal differentiation complex (EDC). Genes in the EDC showed significant changes in expression on the patients’ skin [97].

(b) Concerning the epidermal barrier, genes were associated with increased IgE production, with increased IgE production, or both, such as IL-4 and high-affinity IgE receptors. Skin barrier function may have been impaired due to genetic susceptibility, resulting in elevated chymotrypsin levels in the stratum corneum, leading to premature rupture of corneodesmosomes and epidermal skin barrier dysfunction [98].

(c) The immunological system of eczema focused on T helper type 2 (Th2) cell-derived cytokines, including interleukin (IL)-4 and IL-13, and keratinocyte-derived cytokines, as well as thymic stromal lymphopoietin (TSLP) and IL-33, which contribute to Th2-mediated skin inflammation in AD [99].

(d) One environmental trigger of eczema is microbial exposure (*Staphylococcus aureus*, *Staphylococcus epidermidis*, *Streptococcus*, *Propionibacterium*, and *Corynebacterium*). The exacerbation of skin inflammation may be mediated by superantigens, including staphylococcal enterotoxin-A and -B and toxic shock syndrome toxin (TSST)-1, which lead to polyclonal activation and the stimulation of T cells [100]. Climate factors, including temperature, humidity, and UV exposure, can influence the prevalence of eczema. Moderate UV exposure and elevated temperature appear to be protective against the development of eczema, whereas a combination of high humidity and precipitation is associated with an increased risk of eczema [101]. Sweating excessively aggravates eczema symptoms and irritates the skin because of high humidity. This also promotes the growth of dust mites and mold. Similarly, precipitation can trigger flare-ups of eczema, perhaps due to reduced UV exposure, cooler temperatures, and increased time spent indoors with potentially drying heating systems.

### 4.2. Pharmacological Effects on Eczema

Western and Chinese medicines were suitable for managing eczema symptoms, especially during flare-up periods.

Since Western medicines can act as corticosteroids, immunomodulators, and moisturizers, these may reduce inflammation, modify and suppress immune response, and hydrate the skin and repair the skin barrier. However, the most common side effects are skin thinning, adrenal suppression, skin irritation, increased risk of infections, skin irritation, and allergic reactions [102].

The usage of Chinese medicines to treat eczema symptoms may focus on “Qi”, and the main pharmacologic factors include “wind”, “dampness”, and “heat”, according to Chinese medicine theory [103]. “Qi” is often translated as “vital energy,” “life force,” or “material force”, which sustains physical health, emotional balance, and spiritual vitality, while “wind”, “dampness”, and “heat” are the metaphorical concepts used to describe patterns of disease and imbalance in the body, often influenced by environmental factors.

### 4.3. Pharmacological Functions for Eczema

Western medicines have (a) anti-inflammatory, (b) antipruritic, (c) vasoconstrictive, (d) immunosuppressive, (e) antimitotic, and (f) antiproliferative properties for eczema:

(a) This factor is bound to the glucocorticoid receptor, leading to downstream effects such as the inhibition of phospholipase A2, NF-kappa B, etc., to prevent inflammation [104];

(b) This factor targets the H1 receptor, neuropeptides (e.g., substance P, nerve growth factor), or cytokines (e.g., IL-31) for some types of itching [105];

(c) This factor mediates vasoconstrictors, such as angiotensin-II, arginine-vasopressin, endothelin-1, and noradrenaline, to regulate the blood flow [106];

(d) This factor suppresses the transcription of T-cell cytokine genes, IL-2, IL-3, IL-4, granulocyte macrophage colony-stimulating factor, TNF-α, and IFN-γ expression [107];

(e) This factor inhibits cell division and proliferation to eliminate excessive or abnormal skin cell growth [108];

(f) This factor inhibits fibroblast proliferation, reducing mast cell numbers through the glucocorticoid on the skin [109].

Chinese medicines mainly have their anti-inflammatory properties, as discussed above with regard to the pharmacologic factors. Generally, these mitigate the effects of AD by restoring the skin barrier through balancing Th1/Th2 cell levels, regulating the expression of cytokines and chemokines [110].

### 4.4. Pharmacological Mechanism of Western Medicines

Topical corticosteroids modify and regulate the functions of epidermal and dermal cells and lead leukocytes to participate in proliferative and inflammatory skin diseases. After crossing the cell membrane, they react with receptor proteins in the cytoplasm to form a steroid receptor complex. The steroid receptor complex further enters the nucleus and binds to DNA, altering the transcription of messenger RNA (mRNA) for protein synthesis. Topical corticosteroids play an important role in both the stimulation and inhibition of specific glycoproteins. Lipocortin is a glycoprotein that inhibits phospholipase A2 and releases arachidonic acid from phospholipids as a precursor of prostaglandins and leukotrienes. The main purpose of using topical corticosteroids is to inhibit mRNA for the formation of interleukin-1, causing eczema [111].

Non-steroidals are usually PDE4 inhibitors, which increase the intracellular cyclic adenosine monophosphate (cAMP) level. This is a negative regulator of pro-inflammatory cytokines, which causes a decrease in the inflammatory mediator’s response, such as IL-4 and IL-13, to prevent eczema [112].

### 4.5. Pharmacological Mechanism of Chinese Medicines

Eczema is mainly associated with cytokine–cytokine receptor interactions, tumor necrosis factor (TNF), mitogen-activated protein kinase (MAPK), the nuclear factor kappa-light-chain-enhancer of activated B cells (NF-κB), toll-like receptors, T-cell receptors, and type 1 T helper cell (Th1) and type 2 T helper cell (Th2) differentiation pathways. Chinese medicines such as *Coptis chinensis* Franch, *Phellodendron chinense* Schneid, *Angelica sinensis* (Oliv.) Diels, *Rehmannia glutinosa* Libosch, *Curcuma longa* L., and sesame oil usually downregulate the mRNA expression of mitogen-activated protein kinase 14 (MAPK14), RELA proto-oncogene, NF-KB subunit (RELA), T-box transcription factor (T-bet), and GATA binding protein 3 (GATA3) to reduce the response levels of immunoglobulin E (IgE) and interleukin 4 (IL-4), thereby reducing the response to inflammatory mediators [113].

### 4.6. Pharmacological Mechanisms that Differ between Western and Chinese Medicines

Basically, the mechanisms of Western and Chinese medicines are similar, as both focus on the immune system to treat eczema. They involve the binding of tumor necrosis factor-α (TNF-α) to its receptor to activate two intracellular signaling pathways, namely mitogen-activated protein kinase (MAPK) and nuclear factor kappa-light-chain-enhancer of activated B cells (NF-κB). These pathways are usually downregulated at the level of interleukins to mRNA expression for modulating and correcting the immune system to prevent and fight against eczema. Chinese medicines focus on the root cause versus symptoms with a detailed diagnosis system for the whole body, mind, and spirit.

However, Western medicines are topical corticosteroids and non-steroidals applied to reduce or modulate the symptoms of eczema. These cannot be used in the long term, because they may cause local and systemic adverse effects on the infected skin area. Local adverse reactions include atrophy, striae, rosacea, perioral dermatitis, acne, and purpura, and less frequent adverse reactions include hirsutism, pigmentation changes, delayed wound healing, and exacerbated skin infections. Systemic adverse effects consist of hyperglycemia, glaucoma, and adrenal insufficiency [89]. Thus, integrated Chinese and Western medicine (ICWM) therapy has been applied to compensate for these inadequacies. It is a collaborative approach that combines the strengths of Traditional Chinese Medicine (TCM) and Western medicine to achieve more holistic and effective healthcare. Western medicine (WM) is considered biomedicine, conventional medicine, or mainstream medicine in the healthcare system.

### 4.7. Integrated Chinese and Western Medicine Therapy

A systematic review and meta-analysis on integrated Chinese and Western medicine interventions for atopic dermatitis reported that CM is widely used for the management of AD in China and is used in combination with conventional therapy, such as ICWM. It also identified ICWN processing as leading to a better improvement in the severity of AD than applying WN alone [114], but with an increased risk of adverse events [107]. Based on clinical research, the effective rate of ICWMs was 88%, which was greater than that of the WM (70%) used to treat eczema [114].

Growing evidence has shown that ICWMs have good curative effects on eczema. Chinese medicines are concerned with regulating the immune function and boosting mental health. Functions and indications include clearing away heat and dampness, relieving itching, strengthening the spleen, and activating blood circulation. Compared to Western medicine, the medical properties of Chinese medicines tend to be milder and cannot achieve immediate action. However, Western medicines reduce the risk of eczema symptoms quickly, which complements Chinese medicine in combined therapy. These are the best approaches for ICWM treatments against eczema, as used in recent research studies.

### 4.8. Chinese and Western Medicinal Ointment Usage

The usage of Chinese and Western medicinal ointments is essential to fight against eczema. Usually, Chinese and Western medicinal ointments are applied topically 1 to 3 times per day to the infected area. No matter whether it is a Chinese or Western medicinal ointment, it should be used at the right time after diagnosis, thinking about “safety”, “quality”, “effectiveness”, “dosage”, “frequency” and “function”, because Chinese medicinal ointments are always a mixture of herbs. Different ratios of herbs may affect the therapeutic effects. Western medicine has unique ointments, and their purity is comparatively high. The effectiveness of Western medicinal ointments is greater than that of Chinese medicinal ointments, especially when using topical corticosteroids and non-steroidal agents, due to their purity and potency, though side effects, as mentioned above, are inevitable.

### 4.9. Chinese and Western Medicinal Ointment Bases

The base of the ointment is a vital factor in assessing the effectiveness of medications, particularly in dermatological applications, as it influences the rate of drug release, skin penetration and absorption, hydration and occlusion, therapeutic effectiveness, and tolerability. In Western medicine, petrolatum-based (lipophilic) ointments are utilized, which contain occlusive agents that create a barrier to minimize trans-epidermal water loss and safeguard skin hydration. Moisturizing creams incorporate humectants, such as glycerin or urea, which draw water into the stratum corneum. This action enhances the thickness of the epidermis, alleviating inflammation and improving barrier proteins like filaggrin and loricrin. Absorbents are water-based formulations designed to manage skin moisture, thereby sustaining a balanced hydration environment. Conversely, Chinese medicines often employ natural, plant-derived carriers, following TCM’s holistic approach, such as sesame oil. This oil is abundant in antioxidants, is readily absorbed, promotes herb penetration, and nourishes the skin. The selection of the base is made according to its physical characteristics and energetic and therapeutic harmony with the herbal components it conveys.

## 5. Conclusions

Chinese medicinal ointments commonly contain *Coptis chinensis* Franch, *Phellodendron chinense* Schneid, *Angelica sinensis* (Oliv.) Diels, *Rehmannia glutinosa* Libosch, *Curcuma longa* L., and sesame oil, while Western medicinal ointments include topical corticosteroids and non-steroidal agents, such as hydrocortisone, desonide, clobetasol propionate, fluocinolone, mometasone, fluticasone, aclometasone, prednicarbate, prednisone, prednisolone, triamcinolone acetonide, and crisaborole. The active ingredients in Chinese and Western medicinal ointments have advantages and disadvantages for treating eczema and when acting as skin care products, although they process similar mechanisms and pathways. They are being refined to ensure repeatability, scientific rigor, and compatibility with precision medicine. The effectiveness of Western medicinal ointments is greater than that of Chinese medicinal ointments, especially when using topical corticosteroids and non-steroidal agents, due to their purity and potency. However, side effects, as mentioned above, are inevitable. ICWMs have good curative effects on eczema. Chinese medicines are concerned with regulating the immune function and boosting mental health. This is a milder approach and cannot achieve immediate action. However, Western medicines reduce the risk of experiencing eczema symptoms quickly, which complements Chinese medicine in combined therapy.

## 6. Future Aspects

Proper use of Chinese and Western medicinal ointments is another important criterion. Much more work needs to be carried out, particularly on the Chinese medicinal ointments with different formulations that need to undergo safety assessments for the treatment of eczema. However, these assessments should follow Clinical Practice Guidelines (CPGs) that blend diagnostic and therapeutic approaches from both systems.

## Figures and Tables

**Table 1:** The guidelines of the American Academy of Dermatology on eczema [11].

Features	Conditions
Essence	▪ Pruritus; ▪ Acute, subacute, and chronic; ▪ Chronic or relapsing history; ▪ Typical morphology and age-specific patterns; ▪ Facial, neck, and extensor in infants and children; ▪ Current or previous flexural lesions at any age; ▪ Sparing of the groin and axillary regions.
Important	▪ Early age of onset; ▪ Atopy; ▪ Personal and/or family history; ▪ Raised IgE levels; ▪ Xerosis (dry skin).
Association	▪ Atypical vascular responses; ▪ Facial pallor, white dermatographism (delayed blanch response); ▪ Keratosis pilaris/pityriasis/alba/hyperlinear palms/ichthyosis; ▪ Ocular/periorbital changes; ▪ Other regional findings; ▪ Perioral/periauricular lesions; ▪ Perifollicular accentuation/lichenification/prurigo lesions.
Exception	▪ Other inflammatory dermatoses; ▪ Ichthyoses; ▪ Infections and infestations; ▪ Immunodeficiencies; ▪ Immunological disorders; ▪ Malignancies; ▪ Metabolic disorders; ▪ Urticaria pigmentosa, Epidermolysis bullosa pruriginosa.

**Table 2:** Common topical corticosteroids and non-steroidal agents used in Western medicine against eczema.

	Name	Chemical Structure	Function(s)	Mechanism(s)
Topical corticosteroids are essential treatments for inflammatory skin conditions and are available in formulations such as ointments, creams, lotions, gels, foams, oils, solutions, and shampoos [13].	Hydrocortisone	17-alpha-hydroxy-C21-steroid [14] 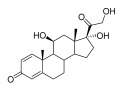	Anti-inflammatory	Reduces and attenuates interleukin (IL)-1β, IL-8, and IL-6 levels in response to tumor necrosis factor-α (TNF-α)-induced inflammatory responses, suppressing the production of cyclooxygenase-2 (COX-2) and inducible nitric oxide synthase (iNOS) [15].
	Desonide	11-beta-hydroxy-C21-steroid [16] 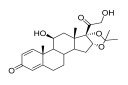	Anti-inflammatory, antipruritic, and vasoconstrictive	Interacts with glucocorticoid receptors in the cytoplasm of skin cells, which bind to specific DNA sequences and suppress the production of pro-inflammatory cytokines, leading to a decrease in the skin’s inflammatory response [17]. Reduces the blood flow narrows the infection area of the blood vessels, as well as prevents the delivery of inflammatory cells and molecules to inflammatory sites [18].
	Clobetasol propionate	17-O-propionate ester steroid [19] 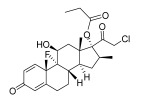	Anti-inflammatory, immunosuppressive, and antimitotic properties	Interacts with glucocorticoid receptors in the cytoplasm and penetrate the cell membrane to form a clobetasol receptor complex, as well as downregulate the expression of inflammatory factors and regulate the transcription of various genes through cyclooxygenase-2 (COX-2), leading to increased levels of pro-inflammatory cytokines (such as lipocortin-1) and decreasing the rates of pro-inflammatory prostaglandin and leukotriene synthesis in skin inflammation [20]. Inhibits the functions of immune cells, including T-lymphocytes, macrophages, and dendritic cells, which decreases the levels of inflammatory mediators such as histamine to suppress the activation of immune cells and migration to the infection area [21].
	Fluocinolone	6-alpha-hydroxy-C21- fluorinated steroid [22] 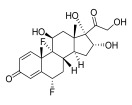	Anti-inflammatory	Inhibits inflammatory cytokine secretion for the cluster of differentiation 14 (CD14), macrophage colony-stimulating factor (M-CSF), macrophage inflammatory protein 3α (MIP-3α), and tumor necrosis factor-alpha (TNF-α) [23].
	Mometasone	11-beta-hydroxy-C21- chlorinated steroid [24] 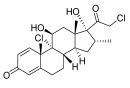	Anti-inflammatory and antiproliferative	Downregulates the inflammatory gene expression level and inhibits the transcription of genes encoding pro-inflammatory cytokines, chemokines, adhesion molecules, and enzymes, including cyclooxygenase-2 (COX-2) and inducible nitric oxide synthase (iNOS) [25].
	Fluticasone	11-beta-hydroxy-C21- trifluorinated steroid [26] 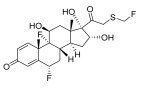	Anti-inflammatory	Inhibits the proliferation of anti-CD3-induced human T-lymphocytes, attenuates tumor necrosis factor-alpha (TNF-α)-induced endothelial cell adhesion molecule expression, and suppresses interleukin-5 (IL-5)-induced blood eosinophilia and IL-5- or platelet-activating factor-stimulated eosinophil accumulation on the skin [27].
	Aclometasone	17-O-dipropionate ester steroid [28] 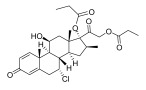	Anti-inflammatory, antipruritic, and vasoconstrictive properties	Induces the phospholipase A_2_ inhibitory proteins known as “lipocortins” and regulates inflammatory mediators, including prostaglandins and leukotrienes. Binds to the corticosteroid receptor and migrates to the nucleus, which enhances and represses various genes in the inflammatory pathways [29].
	Prednicarbate	11-beta-hydroxy-C21-ester steroid [30] 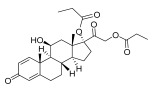	Anti-inflammatory	Inhibits the interleukin 1-alpha cytokine within keratinocytes and suppresses the proliferation of IL-1a in fibroblasts, as well as collagenase induction and IL-6 synthesis, to moderate skin thickness [31].
Non-steroidals are a group of medications commonly used to treat pain and reduce inflammation for muscle and joint pain or swelling, such as gels, creams, or patches [32].	Prednisone	17-alpha-hydroxy-C21-steroid [33] 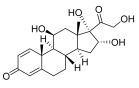	Anti-inflammatory	Inhibits the migration of polymorphonuclear leukocytes and reverses increasing capillary permeability to decrease the inflammation that enters the nucleus and binds to, as well as activates, specific nuclear receptors, resulting in altered gene expression and inhibiting the production of pro-inflammatory cytokines [34].
	Prednisolone	11-beta-hydroxy-C21-steroid [35] 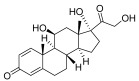	Anti-inflammatory	Same as prednisone.
	Triamcinolone acetonide	11-beta-hydroxy-C21-steroid [36] 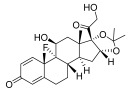	Anti-inflammatory	Inhibits the phospholipase A2 enzyme on the phospholipid layer of the cell membrane, which hinders the decomposition of leukocyte lysosomal membranes and prevents the formation of arachidonic acid to cause the expression of cyclooxygenase (COX) and lipoxygenase (LOX) [37]. Inhibits the nuclear factor kappa-B (NF-κB), causing a decrease in the protein expression levels of interleukin-6 (IL-6), interleukin-8 (IL-8), monocyte chemoattractant protein-1 (MCP-1), and cyclooxygenase-2 (COX-2) [38,39].
	Crisaborole	5-hydroxy-1,3-dihydro-2,1- benzoxaborole [40] 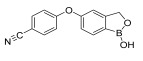	Anti-inflammatory	Facilitates skin penetration and binds to the bimetal center of the phosphodiesterase-4 enzyme, which regulates cyclic adenosine monophosphate in cells and produces cytokines [41].

**Table 3:** Clinical studies of topical corticosteroids and non-steroidal ointments.

	Name	Dosage (%)	Side Effects	Efficiency and Effectiveness
Topical corticosteroids	Hydrocortisone	0.1	Burning or stinging, dryness or flaking, itchiness or irritation, and skin discoloration.	One hundred ninety-four adults with clinically diagnosed atopic dermatitis were randomized for treatment with 0.1% hydrocortisone buteprate cream or placebo. There was a significant improvement from 0.1% hydrocortisone buteprate cream in atopic dermatitis patients, and most adverse effects were mild to moderate, including a burning sensation, with 4% of patients using a placebo and only 2% of patients using hydrocortisone buteprate [42].
	Desonide	0.05	Dryness or peeling, itching or irritation, redness or rash, and discoloration.	One hundred thirteen children were enrolled in a multicenter, randomized, investigator-masked, parallel-group study. Patients with a mean age of 4.8 years and mild-to-moderate atopic dermatitis were treated with a desonide ointment. This had greater efficacy and led to rapid improvement, as well as being safer than the 1% hydrocortisone ointment and suitable for up to 6 months of use [43].
	Clobetasol propionate	0.05	Burning or stinging, itching, dryness, irritation, redness, rash, folliculitis, cracking or peeling skin, and changes in skin pigmentation.	One hundred thirteen adults with eczema were evaluated for the clinical response severity of specific signs and symptoms. Overall, 4% of patients treated with clobetasol experienced drug-related reactions, and 6% of patients treated with clobetasol experienced drug-related reactions, which were resolved within 1 week after the end of treatment [44].
	Fluocinolone	0.01	Burning, stinging, or itching, dryness or peeling, redness or irritation, folliculitis, and skin discoloration.	Sixty-six otic eczema patients were treated with fluocinolone acetonide twice daily for 7 days and 8 days of follow-up to study the changes in otoscopic signs, such as erythema, oedema, and scaling. Fluocinolone led to significant improvement in patients. It was an effective and safe treatment for otic eczema [45].
	Mometasone	0.1	Burning or stinging, dryness or redness, itching or irritation, acne-like bumps, and changes in skin pigmentation.	One hundred and twenty patients with chronic hand eczema were treated with mometasone furoate fatty cream daily until the dermatitis subsided or for a maximum of 9 weeks, indicating that intermittent use of mometasone furoate fatty cream for the treatment of chronic hand eczema was safe and effective [46].
	Fluticasone	0.05	Burning or stinging, dryness or irritation, redness or itching, skin rash or hives, and changes in skin pigmentation.	Thirty-two children aged 3 months to 6 years old, with moderate-to-severe atopic dermatitis, were treated with 0.05% fluticasone propionate cream twice daily for 3 to 4 weeks. It can safely treat severe eczema in children aged 3 months and older for up to 4 weeks [47].
	Aclometasone	0.05	Itching, burning, or stinging, dryness or irritation, redness or erythema, papular rashes, lightened skin color, and folliculitis.	Thirty-nine children with eczema were treated with 0.05% alclometasone dipropionate cream during a three-week open study. There was a good overall response, with 28 children (72%) experiencing complete resolution of monitored signs and symptoms, as well as seven children (18%) experiencing marked or moderate improvement in monitored signs and symptoms [48].
	Prednicarbate	0.05	Burning, itching, or stinging; dryness, scaling, or cracking; redness or irritation; folliculitis, acne or pimples; and increased hair growth.	Twenty-four healthy volunteers applied 0.25% prednicarbate cream or the corresponding vehicle to one forearm and 0.1% betamethasone-17-valerate cream or 0.05% clobetasol-17-propionate cream to the other forearm twice daily in a double-blind controlled trial. Normally, 0.1% betamethasone-17-valerate and 0.25% prednicarbate cream were reported to be about equipotent in the treatment of atopic eczema, and the latter preparation had an increased ratio between its desired anti-inflammatory effect and its unwanted anthropogenic effect [49].
**Non-steroidals**	**Name**	**Dosage (%)**	**Side effects**	**Efficiency and effectiveness**
	Prednisone	0.01	Rashes, thinning of the skin (atrophy), increased susceptibility to bruising, delayed wound healing, acneiform eruptions, and stretch marks (striae).	One hundred and twenty skin lesion patients received prednisone for six months to ten years. Warts and herpes zoster cases increased with treatment duration, but the total number of skin manifestations did not increase significantly [50].
	Prednisolone	0.05	Skin thinning (atrophy), stretch marks (striae), acne or acne-like eruptions, delayed wound healing, increased facial puffiness, and hair loss or increased hair growth.	Thirty-eight patients with severe eczema were randomly assigned to receive either prednisolone for 2 weeks, followed by a placebo for 4 weeks, or cyclosporine for 6 weeks, followed by 12 weeks of treatment. The results showed that cyclosporine was significantly more effective than prednisolone in treating severe eczema in adults [51].
	Triamcinolone acetonide	0.1	Itching, burning, or stinging, dryness or irritation, redness or rash, pale or lightened skin, acne or pimples, and increased hair growth	Eighteen eczema patients were treated with triamcinolone acetonide (TA) cream of 0.1% and a cream containing 0.1% TA plus 0.025% retinoic acid (RA) for one to three weeks. The results indicated that the addition of 0.025% RA to medium-potency topical steroids did not abolish the anti-inflammatory properties of the latter and that inflamed skin could tolerate this combination [52].
	Crisaborole	2	Application site burning or stinging, redness or irritation, and itching (pruritus).	Two identically designed, vehicle-controlled, double-blind studies enrolled and randomized (2:1, crisaborole: vehicle) patients aged 2 years or older with an Investigator’s Static Global Assessment (ISGA) score of mild or moderate for twice-daily use for 28 days. The results of crisaborole with a favorable safety profile demonstrated improvements in all efficacy measures, including overall disease severity, pruritus, and other symptoms of atopic dermatitis [53].

**Table 4:** Selected Chinese medicinal plants used against eczema.

	Name	Major Active Ingredients and Chemical Structure	Function(s)	Mechanism(s)
**Chinese Medicinal plants**	*Coptis chinensis* Franch. (Huang Lian)	(a) berberine 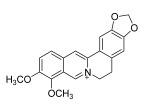 (b) epiberberine 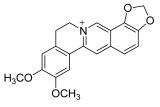 (c) palmatine 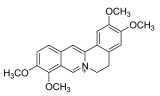 (d) coptisine [54] 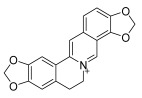	Anti- inflammatory	(a) Inhibiting eotaxin and pro-cytokine inflammation, as well as the improvement of atopic dermatitis (AD) by downregulating eukaryotic translation initiation factor 3 subunit F (EIF3F) and mucosa-associated lymphoid tissue lymphoma translocation 1 (MALT1) [55]. (b) Inhibiting serum levels of pro-inflammatory cytokines and activating mitogen-activated protein kinases to prevent inflammation [56]. (c) Increases the expression of microtubule-associated proteins 1A/1B light chain 3B (LC3), Beclin-1, phospho-LKB1, phospho-AMPK, autophagy (Atg) protein 5, Atg12, and Atg5-Atg12, while P62 and ribosomal protein S6 kinase B1 (p-p70S6K1) expression is decreased to improve the inflammatory factors [57]. (d) Inhibits the production of the pro-inflammatory cytokines interleukin-1β (IL-1β) and interleukin-6 (IL-6) by regulating the expression of cytokine mRNA and suppresses LPS-stimulated inflammation by blocking the activation of nuclear factor-κB (NF-κB), mitogen-activated protein kinase (MAPK), and phosphoinositide-3-kinase (PI3K) / Akt in macrophages [58].
	*Phellodendron chinense* Schneid (Huang Bai)	(a) berberine (same as above) (b) phellodendrine 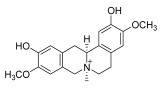 (c) palmatine (same as above) [59]	Anti- inflammatory	(b) Downregulating the expression level of p65 mRNA to reduce the release of inflammatory cytokines, such as tumor necrosis factor-α (TNF-α) and interleukin-1β (IL-1β), to relieve lumbar disc pain [60].
	*Angelica sinensis* (Oliv.) Diels	ligustilide [61] 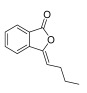	Anti- inflammatory	Exhibits anti-inflammatory activities by blocking the activation of mitogen-activated protein kinases (MAPKs)/IκB kinase (IKK), transcription factor activator protein-1 (AP-1), and nuclear factor-kappa B (NF-κB), as well as decreasing the intracellular reactive oxygen species (iROS) [62].
	*Rehmannia glutinosa* Libosch	Iridoid glycosides (a) catalpol 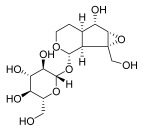 (b) aucuboside 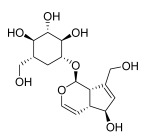 Phenylpropanoid glycoside (c) acteoside [63] 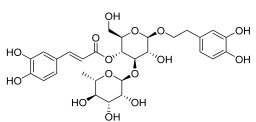	Anti- inflammatory	(a) Alleviates skin lesions and mast cell infiltration via the regulation of pro-Th2 and Th2 cytokines in vivo, which also reduces the level of immunoglobulin E (IgE) in atopic disease [64]. (b) Moderates colitis symptoms, such as weight loss, a high disease activity index, and inflammatory response, through the inhibition of IL-17 expression in Th17 cells [65]. (c) Inhibits the β-catenin/CTGF signaling pathway to downregulate the expression levels of the fibrin-related proteins β-catenin, CTGF, α-SMA, collagen III, and HSP47 [66].
	*Curcuma longa* L.	Curcumin [67] 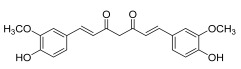	Anti- inflammatory	Alleviates ovalbumin-induced atopic dermatitis and associated asthma symptoms in mice by restoring skin pathology, inhibiting inflammatory cell infiltration and cytokine expression, and restoring redox and NF-κB signaling balance [68].
	Sesame oil	Sesamin [69] 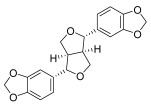	Anti- inflammatory	Suppresses the expression levels of pro-inflammatory cytokines (TNF-α, IL-1β, IL-6) that induce cancer cell apoptosis and autophagy through multiple signaling pathways, such as NF-κB, JNK, p38 MAPK, PI3K/AKT, caspase-3, and p53 [70].

**Table 5:** Clinical studies of selected Chinese medicinal ointments.

	Name	Dosage (%)	Side Effects	Efficiency and Effectiveness
**Chinese Medicinal plants**	*Coptis chinensis* Franch. (Huang Lian)	5–15	Allergic reactions, skin irritation, and photosensitivity.	Eight-week-old guinea pigs were randomly divided into six groups, with ten in each group. Biostir-AD^®^ (Biostir, Kobe, Japan) was applied to the back of each guinea pig once every 3 days for 2 weeks to induce eczema-like skin lesions. Huang Lian ointment quantities of 0.45, 0.90, and 0.18 g/guinea pig were applied once a day; hydrocortisone at 50 mg/guinea pig was used as the positive control group; and sesame oil emulsion without Huang Lian ointment was used as the negative control group and model group. The results showed that Huang Lian ointment repaired the skin barrier by affecting the expression levels of c-Jun, JunB, and filaggrin and suppressed the inflammatory response by inhibiting the AGE-RAGE signaling pathway, thereby treating eczema in vivo and in vitro [71].
	*Phellodendron chinense* Schneid (Huang Bai)	5–15	Mild irritation, contact dermatitis, and photosensitivity.	Atopic dermatitis patients were selected from the diagnostic clinic of Dongzhimen Hospital according to the severity index (EASI) guidelines, and 6 to 8-week-old BALB/c female mice were used as the 1-Chloro-2,4-dinitrobenzene (DNCB)-induced atopic dermatitis model, receiving Huang Bai ointment on local skin lesions 3 to 4 times per day. The results demonstrated that Huang Bai ointment was a suitable treatment since it mitigated skin inflammation in atopic dermatitis patients and the 1-Chloro-2,4-dinitrobenzene (DNCB)-induced atopic dermatitis mouse model by reinvigorating the T-cell immune balance to decrease the mRNA expression of pro-inflammatory cytokines, including IL-1β, TNF-α, IL-17, IL-4, and IL-13 [72].
	*Angelica sinensis* (Oliv.) Diels (Danggui)	5–10	Contact dermatitis, photosensitivity, and skin irritation.	*Angelica sinensis* (Oliv.) Diels was topically applied to the dorsal skin of 1-Chloro-2,4-dinitrobenzene (DNCB)-challenged mice for 11 days. The result identified that *Angelica sinensis* (Oliv.) Diels reduced the levels of cytokines (IL-4, IL-6, TNF-α, and IFN-γ) and the expression levels of NF-κB, phosphor-IκBα, and phosphor-MAPKs to modulate pruritus and inflammation in atopic dermatitis [73].
	*Rehmannia glutinosa* Libosch (Di huang)	5–10	Contact irritation, allergic reactions, photosensitivity, dryness, and tingling.	Atopic dermatitis was induced by spreading an atopic-inducing reagent, biostir atopic dermatitis (Biostir Inc., Japan), which is a natural chemical found in mites, on the mice’s skin for 3 weeks. Then, 100 mg of Di huang ointment was applied to the shaved back skin twice a week for 3 weeks. The results indicated that it inhibited the expression of cytokines, chemokines, and adhesion molecules and subsequently blocked the accumulation of leukocytes, which may be responsible for its inhibitory effect on atopic dermatitis-like skin lesions [74].
	*Curcuma longa* L.	1–5	Allergic contact dermatitis, skin staining, and photosensitivity.	One hundred and fifty eczema patients were treated with *Curcuma longa* L ointment twice daily for 4 weeks, and the results indicated that this approach reduced eczema symptoms by 28% to 35%. The cream for four patients complaining of mild burning at the application site was well tolerated, and only one patient reported hyperpigmentation [75].
	Sesame oil	5–20	Allergic reactions, comedogenic risk, and photosensitivity.	Forty women with breast cancer who were undergoing five weeks of radiation therapy were randomly divided into two groups: twenty with sesame oil and twenty with a placebo. According to this research, sesame oil may treat acute dermatitis caused by radiation therapy [76].

**Table 6:** Types of Western and Chinese medicines, their treatment durations, and their side effects.

Types of Western and Chinese Medicines	Name	Duration	Most Severe Symptom
Topical corticosteroids	Hydrocortisone	More than 1 day used single	Absorb into the systemic circulation
	Clobetasol propionate	Long-term usage	Skin atrophy
	Fluocinolone	Long-term usage	Rebound inflammation
	Mometasone	Long-term usage	Skin atrophy
	Fluticasone	Long-term usage	Adrenal gland problems
	Alclometasone	Long-term usage	Breathing problems
	Prednicarbate	Several weeks	Allergic contact dermatitis
Non-steroidals	Prednisone	Long-term usage	Hyperglycemia
	Prednisolone	Long-term usage	Leucocyte infiltration and activation problems
	Triamcinolone acetonide	Long-term usage	Skin atrophy
	Crisaborole	Long-term usage	Skin atrophy
Chinese medicine	Berberine	Long-term usage	Skin to darken
	Phellodendrine	Long-term usage	Skin allergy
	Ligustilide	Long-term usage	Skin poisoning
	Catalpol	Long-term usage	Blood-brain barrier
	Aucuboside	Long-term usage	Skin poisoning
	Acteoside	Long-term usage	Hypertension

## Data Availability

No new data were created or analyzed in this study.
